# Standardization of the psychometric hepatic encephalopathy score in a French population

**DOI:** 10.1371/journal.pone.0257136

**Published:** 2021-09-10

**Authors:** Olivier A. Coubard, Kinga M. Ober, Marie Gaumet, Marika Urbanski, Jean-Noël Amato, Vincent Chapron, Nicolas Weiss, Kiyoka Kinugawa, Karin Weissenborn, Dominique Thabut

**Affiliations:** 1 The Neuropsychological Laboratory, CNS-Fed, Paris, France; 2 Service de Médecine et de Réadaptation Gériatrique et Neurologique, Hôpitaux de Saint-Maurice, Saint-Maurice, France; 3 Department of Neurology & Institut de Cardiométabolisme et Nutrition, Neurological Intensive Care Unit, Groupe Hospitalier Pitié-Salpêtrière, AP-HP, Sorbonne Université, Paris, France; 4 Brain Liver Pitié-Salpêtrière (BLIPS) Study Group, Groupe Hospitalier Pitié-Salpêtrière, AP-HP, Sorbonne Université, Paris, France; 5 Centre de Recherche Saint-Antoine, UMR_S 938 INSERM-Sorbonne Université, Paris, France; 6 Functional Explorations and Sleep Investigation Unit for Older Patients, AP-HP, Ivry-sur-Seine, France; 7 Biological Adaptation and Aging, UMR 8256, CNRS-Sorbonne Université, Paris, France; 8 Department of Neurology, Hannover Medical School, Hannover, Germany; 9 Service d’Hépato-gastro-entérologie, Groupe Hospitalier Pitié-Salpêtrière, AP-HP, Sorbonne Université, Paris, France; Medizinische Fakultat der RWTH Aachen, GERMANY

## Abstract

The Psychometric Hepatic Encephalopathy Score (PHES) has previously been standardized in thirteen countries on three continents, confirming its status of gold standard test to detect minimal hepatic encephalopathy (MHE). In the meantime, performance has also been shown to vary with variables such as age, education, and barely sex. The present study aimed at standardizing the PHES in a French population. One hundred and ninety-six French healthy participants completed a French version of the paper-and-pencil PHES, involving five tests and six measures. Importantly, the balance was perfect between all levels of the three controlled factors, which were sex, age (seven decade-levels from 20–29 to 80–89 years), and education (two levels below or above 12 years of education). Raw measures were transformed to fit the normal distribution. ANOVAs on transformed variables showed no effect of sex, but an effect of age on all measures, and of education on five measures. Multiple or simple regressions were completed to build up normograms. Thorough analysis of variability within each test failed to find outliers that may bias the results. Comparison between French and seminal German data showed that they highly fitted though cultural and cognitive style specificities could be observed. This is the first study to standardize the PHES in a French population and to extensively explore the effects of sex, age and education using perfectly balanced samples. Subtle differences between countries of the same continent emphasize the need to build up normative data in each country to get accurate PHES in patients.

## Introduction

The tight relationship between brain and body has not been more elegantly illustrated than in hepatic encephalopathy (HE), a dramatic brain condition as an effect of liver failure. HE is a neurological and psychiatric syndrome caused by liver insufficiency and/or portal-systemic blood shunting [1]. Though studied for 170 years [2], its pathogenesis remains complex [3]. Amid the potential phenomena, hyperammonemia associated with systemic inflammation plays a critical role [2, 4]. With hepatic failure, ammonia accumulates in the systemic circulation. In chronic liver disease on the one hand, it yields astrocytic and neuronal dysfunction and thereby HE. In patients without chronic liver disease displaying acute liver failure on the other hand, the clinical presentation is also governed by the development of brain edema which results from increased levels of glutamine, inflammatory cytokines and lactate [2]. The inherent taxonomy describes four types of HE: A, acute liver failure; B, porto-systemic shunts; C, liver cirrhosis and porto-systemic bypass; and D, acute-on-chronic liver failure [1]. Clinically, HE may be overtly detectable in the examination as global, but unspecific, neurological deficits ranging from movement to personality disorders, thus needing exclusion of other causes of brain dysfunction [5, 6]. Movements disorders include ataxia, asterixis, bradykinesia, hyperreflexia, hypertonia, rigidity, and tremor, whilst personality disorders may mimic mania with frequent agitation and aggressivity. But none of these symptoms is pathognomonic [1–3]. The so-called overt HE according to the International Society for Hepatic Encephalopathy and Nitrogen Metabolism (ISHEN) corresponds to grades II to IV of the West Haven criteria depending on the patient state from somnolence with disorientation to coma [7]. Overt HE affects cognition in different ways. Attention and executive systems are impaired. Visual perception disorders may take the form of agnosia, macropsia, distortion and hallucinations [5, 6]. Operative criteria have been suggested to be disorientation for time in grade II and disorientation for time and space in grade III [1].

HE can also be clinically undetectable. In this case, neuropsychological and/or neurophysiological examination are needed to detect the so-called covert HE according to the ISHEN [7]. Covert HE embraces grade I of the West Haven criteria and, at a lower level, minimal HE (MHE). In grade I, despite normal orientation, the patient usually exhibits difficulties in attention spans and calculation associated to behavioral issues. In MHE, specific neuropsychological and/or neurophysiological tests allow the examiner to unveil the patient’s difficulties [8, 9]. From the neuropsychological viewpoint, MHE affects three different areas of cognition: attention and executive functions resulting in inattention and dysexecutive difficulties; motor coordination yielding slowness and inaccuracy; and visuospatial perception leading to visuospatial and possibly visual-constructional difficulties [9–16]. In daily life, this neuropsychological syndrome realizes as disinterest, distraction, clumsiness, falls and fatigue, which inevitably impacts critical activities such as driving a car, working or ensuring a decent socioeconomic living [9, 17, 18], resulting in lower quality of life [19, 20]. Furthermore, MHE has been suggested to indicate a high risk of overt HE [21, 22]. For those reasons, MHE should be treated and the development of neuropsychological and/or neurophysiological tests with good metrological qualities for detecting MHE has become a challenging issue in hepatology and neurology [1]. There are now available treatments for MHE like lactulose and rifaximin. Moreover, one differential diagnosis of MHE is mild cognitive impairment, which has a different prognosis regarding cognition and reversibility. In situations where liver transplantation is discussed, an accurate diagnosis for cognitive disorders is of major importance.

Several paper-and-pencil neuropsychological tests are available to detect MHE. The Portosystemic Encephalopathy Syndrome (PSE) test, leading to the Psychometric Hepatic Encephalopathy Score (PHES), has been specifically developed to achieve that goal [1, 23]. In its final form, the PHES gathers five tests: the Digit Symbol Test (DST), the Number Connection Test (NCT) in its parts A (letters) and B (letters and numbers), the Serial Dotting Test (SDT), and the Line Tracing Test (LTT) [23]. Importantly, these tests are inherited from previous existing tests, having each a specific goal: DST from the Wechsler Adult Intelligence Scale, WAIS [24], NCT-A and NCT-B from the Trail Making Test [25], SDT and LTT from the Motor power series by Schoppe [26] themselves inspired by Fleishman [27]. As an alternative, the ISHEN has also recommended [28] the Repeatable Battery for the Assessment of Neuropsychological Status exploring attention, visual perception, long-term memory and language [29], though the two latter functions are spared in MHE. For facility or copyright issues, some tests have been used in isolation [30]: DST, NCT-A and B, the Block Design Test and the Digit Span Test [24]–not to be confused with DST–and the Stroop Test [31]. Computerized neuropsychological tests also aim at aiding MHE diagnosis such as the Inhibitory Control Test [32], the Cognitive Drug Research battery [33], the EncephalApp Stroop test [34], and the Continuous Reaction Time test [35]. These tests have in common to assess reaction times, attention and executive control, but precision and visual perception are little involved compared to the said paper-and-pencil tests [3, 36, 37]. Further tests, such as bimanual coordination, d2, and Symbol Digit Modalities Test, have been introduced to refine the detection of MHE [38–40]. In neurophysiology, EEG [41], the critical flicker frequency (CFF) test [42], and evoked potentials [43] have been extensively studied for the detection of MHE, with the CFF emerging as the easiest tool to implement in clinical practice [1].

Since the seminal study by Weissenborn et al. [23], the PHES has proved to be the gold standard to detect MHE due to its accessibility, low cost, easiness, and most importantly its ability to examine all cognitive functions that are likely to be impaired in MHE (see above) [1, 3, 36]. The only limitation of the PHES, as a direct consequence of assessing all these functions, is to vary with sex (though barely), age, education and culture, which requires to build normative data in each country and/or province [44]. To our knowledge, the PHES or elements of it have been standardized–or the performance in PHES in a significant number of healthy participants has been reported–in fifteen studies of thirteen countries: China (two studies in Hefei, Anhui and Wuhan, Hubei) [45, 46], Cuba [47], Germany [23], Italy [48], India [49], Mexico [50], Poland [51], Portugal [52], Romania [53], Spain [54], South Korea (two studies) [55, 56], Turkey [57], and USA (Arkansas, Ohio and Virginia) [58]. To examine the quality of normograms, Tables 1 and 2 show for each study the number of tests and related scores, the characteristics of healthy participants (Table 1) and of liver cirrhosis patients (type C) without overt HE, and the outcome of MHE among those patients using the given norms (Table 2). Regarding the tests, though some materials may have slightly varied between studies thus explaining possible variations in the results [59], all studies respected the form of the seminal PHES using 5 tests and 6 measures: correct symbols in DST, time in NCT-A, NCT-B and SDT, time and errors in LTT [23]. Only two studies in China [45] and South Korea [56] used 3 tests for, respectively, facility and copyright issues: NCT-A, NCT-B and DST in the former; NCT-A, Digit Span Test and Symbol Digit Modalities Test [60] in the latter (Table 1).

**Table 1:** Literature review of PHES in healthy participants.

Study		Battery		Healthy participants					
Year	Country	Test	Score	N	Sex^R^	Age	Age^R^	Edu	Edu^R^
2001	Germany	5	6	120	0.88	16–76	-	no	-
2006	Spain	5	7	884	1.01	20–80	-	yes	-
2008	Italy	5	5	228	0.95	18–85	1.21	4	2.61
2010	India	5	6	83	2.19	19–78	-	yes	-
2011	Mexico	5	5	743	0.84	51±15	0.86	3	0.76
2011	Portugal	5	5	115	0.64	41±13		yes	
2012	Korea	5	8	200	1	20–69		yes	
2013	China	5	5	146	2.11	37±10		yes	
2013	Poland	5	5	316	0.82	18–79		yes	
2015	China	2	2	843	1.41	16–65		yes	
2016	USA	5	5	308	0.78	21–65		yes	
2016	Romania	5	6	260	0.87	19–78		yes	
2016	Cuba	5	5	520	0.92	18–73	1.04	5	1–1.20
2017	Turkey	5	5	185	0.97	46±10	-	yes	-
2017	Korea	3	3	315	0.89	20–69	-	yes	-
2020	France	5	6	196	1	20–89	1	2	1

Studies on standardization of PHES (or elements of it) or reporting a significant number of healthy participants. N: total number of participants. The factor ratio (Sex^R^/Age^R^/Education^R^) was calculated as the number of participants in the first factor level multiplied by the number of factor levels (Sex = 2, men/women; Age/Education = variable, 1^st^/2^nd^/etc. levels) divided by the total number of participants, so that excessive/insufficient number of participants within the first level yielded ratio respectively above/below 1 (e.g., less men than women led to Sex^R^ <1). If the first ratio was 1 in the first level of a given factor, there was a need to calculate the ratio in the next level until confirmed it was 1 in all levels. If the ratio was not 1, there was no need no calculate further. Elsewhere, Age is either the range or the mean±SD or the mean; Education is either the number of factor levels or the information according to which it was or not controlled (yes/no).

**Table 2:** Literature review of PHES in patients.

Study		Patients					
Year	Country	N	Sex^R^	Age	MHE	HE	Death
2001	Germany	63	1.21	43±15	25%	-	-
2006	Spain	-	-	-	-	-	-
2008	Italy	100	2.44	56±11	25%	-	-
2010	India	104	3.95	48	48%	28%	39%
2011	Mexico	84	0.74	54±10	15%	-	-
2011	Portugal	-	-	-	-	-	-
2012	Korea	160	1.76	55±8	26%	-	-
2013	China	53	16.67	45±8	49%	-	-
2013	Poland	50	1.63	18–81	22%	-	-
2015	China	429	-	16–65	28%	-	-
2016	USA	437	1.78	21–65	37%	35%	-
2016	Romania	106	1.04	22–78	35%	-	-
2016	Cuba	-	-	-	-	-	-
2017	Turkey	60	1.27	54±8	32%	-	-
2017	Korea	32	1.31	56±7	37%		
2020	France	-	-	-	-	-	-

After standardization shown in Table 1, patients’ total number (N), sex and age characteristics are reported. MHE: percentage of N patients showing MHE according to the standardized PHES. HE/Death: percentage of followed-up MHE patients experiencing HE and death. Other notations as in Table 1.

With respect to the scores, the number varied from 6 to either 5 or 7–8 whenever for LTT, time and errors were either replaced by [45, 47, 48, 50–52, 57, 58] or added to their sum or error-weighted time [54] or both [55] (Table 1). Though the number of measures changed the cut-off, it did not change the ability of the test to detect MHE. More important are healthy participants’ characteristics. Indeed, a common error in standardization is to focus on the sample size, recruit as many participants as possible, thereby achieving imbalanced groups of participants in the different factor levels [61]. As shown in Table 1, the factor ratios we calculated in the healthy participants were never perfect, except for the age of one Korean study [55]. In patients, such calculation could not be done except for the sex, as only fragmented information was available [44] (Table 1). As a result, MHE was detected in 22–37% of liver cirrhosis patients throughout nine studies. Only for three studies, it was either much lower, 15% [50], or higher, 48–49% [45, 49], which remarkably fitted sex ratios favoring, respectively, women and men (Table 2). Keeping in mind that age and education are even more influential on PHES, it demonstrates how important it is to inform about those variables [44].

The goal and uniqueness of the present study was threefold. First, we aimed at standardizing PHES in a French population of healthy participants. Second, the study involved a collaboration between two teams of neuropsychology (authors OAC, MU), three teams of neurology (NW, KK, KW), and one team of hepatology (DT). Third, the standardization was performed by building up a perfect ratio for the three controlled factors: sex, age, and education.

## Materials and methods

### Participants

Two hundred and ten French participants were recruited to participate in the study that adhered to the tenets of the Declaration of Helsinki. The study was approved by the French ethics committee “Comité pour la Protection des Personnes Île-de France 8” (n° RCB 2012-A00290-43). The sample size used heuristics. All participants received clear information about the study aims and methods and gave their oral consent for ages 20–64 and written consent for retirement ages 65–89. Fourteen of them were not included due to health issues or for not following the instruction. The final sample was made up with 196 healthy individuals from urban and rural areas of nine departments of France (Côte-d’Or, Deux-Sèvres, Haute-Savoie, Maine-et-Loire, Paris, Sarthe, Seine-Saint-Denis, Val-de-Marne, and Vendée) (Table 3).

**Table 3:** Characteristics of healthy participants.

			Age		Education	
AgeG	EduG	N	Women	Men	Women	Men
20–29	<12	7	26.79±2.46	25.01±3.44	10.71±0.76	11.00±0.00
20–29	>12	7	23.76±3.03	23.87±3.78	14.00±1.41	14.00±2.52
30–39	<12	7	34.14±2.26	33.73±2.21	11.00±0.00	10.57±1.13
30–39	>12	7	35.91±2.75	34.99±4.08	16.00±3.32	16.43±0.79
40–49	<12	7	44.68±2.48	44.75±1.63	11.00±0.00	11.0±0.00
40–49	>12	7	43.72±2.25	44.66±3.77	15.29±2.29	16.57±2.51
50–59	<12	7	53.99±3.39	54.93±2.53	9.29±2.63	10.71±0.76
50–59	>12	7	53.24±2.97	53.28±2.68	15.43±1.81	15.71±1.25
60–69	<12	7	66.19±3.68	63.25±3.55	10.14±2.27	9.00±2.83
60–69	>12	7	64.99±2.91	64.60±3.52	14.14±2.27	16.57±2.07
70–79	<12	7	72.84±1.75	74.20±1.92	7.43±3.05	9.71±2.63
70–79	>12	7	74.56±2.95	72.87±2.59	16.00±1.29	16.00±2.58
80–89	<12	7	83.00±2.08	86.09±2.87	7.00±2.94	6.43±2.57
80–89	>12	7	82.73±1.92	86.44±1.72	13.86±2.41	16.00±1.91

Number (N) and mean±standard deviation of age in years and education in years for the different age (AgeG) and education (EduG) groups.

They were 98 men and 98 women, aged 20–89 years, and had less or more than 12 years of education. Importantly, they were optimally distributed (N = 7) by the 2 sex levels (men, women), by the 7 age levels (20–29, 30–39, 40–49, 50–59, 60–69, 70–79, and 80–89 years), and by the 2 education levels (± 12 years). In other words, the ratio as defined in the Introduction was perfect (equal to 1) for the three factors (Table 1). Handedness was right/left for respectively 186/10 participants, and they all had normal or corrected-to-normal vision.

### Material and procedure

The PHES battery was provided by KW and adapted into French by OAC. Thus, materials and procedure were close to those of the seminal study [23]. In brief, the battery comprised the 5 tests as follows: DST, NCT-A, NCT-B, SDT and LTT. In DST, participants were invited to associate symbols to digits by handwriting according to an always visible model. In NCT-A and B, they were asked to link numbers and numbers/letters in alternation, respectively, by drawing a single trace. In SDT, they had to point the centre of circles by writing. In LTT, they had to draw a single trace in a tortuous labyrinth without touching or exceeding its boundaries. The assessment was completed by neuropsychologists OAC, MU, JNA, VC and trained clinical expert MG under the supervision of OAC. The 5 tests led to 6 measures: correct responses for DST; time in s for NCT-A and NCT-B, which included potential online self- or examiner-corrections; time in s for SDT; time in s and errors for LTT noted LTTt and LTTe, respectively. Before completing the PHES, participants aged 50–89 years (N = 112) completed the Mini-Mental State Examination (MMSE) to check for general cognition [62].

### Data analysis

Paper-and-pencil data of all participants were scored twice by two independent raters. When pooled together, if a disagreement occurred, which happened three times for LTTe due to line tracing in the vicinity of the labyrinth boundaries, data were checked until a consensus emerged.

Data were then digitalized and processed as follows. Measures were transformed/retransformed before/after statistics and normograms were built up under Matlab R2017 (The MathWorks, USA). Statistics for normality tests, analyses of variance, simple or multiple regressions, and correlations were done under Statistica 7.0 (StatSoft, USA) and SPSS 12.0 (SPP Inc., USA). Finally, normograms were coded and implemented online under Adobe (Adobe Inc., USA).

## Results

### Preliminary test and measure transformation

General cognition was normal in the 112 participants over 50 years who completed the MMSE. Indeed, the raw score in MMSE was 27 out of 30 or above, corresponding to percentile 50–90 according to French standard considering age and education [63]. In the PHES, the six measures were transformed as follows: DST responses, NCT-A, NCT-B, SDT times and LLTt into their napierian logarithm; LLTe into its square root. Resulting transformations DST^TR^, NCT-A^TR^, NCT-B^TR^, and LLTt^TR^ were normally distributed according to Kolmogorov-Smirnov (KS) and Shapiro-Wilk tests, SDT^TR^ using KS, and LLTe^TR^ was marginally normal using KS.

### Effects of sex, age and education

Three-way ANOVA with sex (2 levels), age (7 levels), and education (2 levels) as between-participant factors did not show any main effect of sex on the six measures DST^TR^, NCT-A^TR^, NCT-B^TR^, LLTt^TR^, SDT^TR^, and LLTe^TR^.

**Fig 1:**
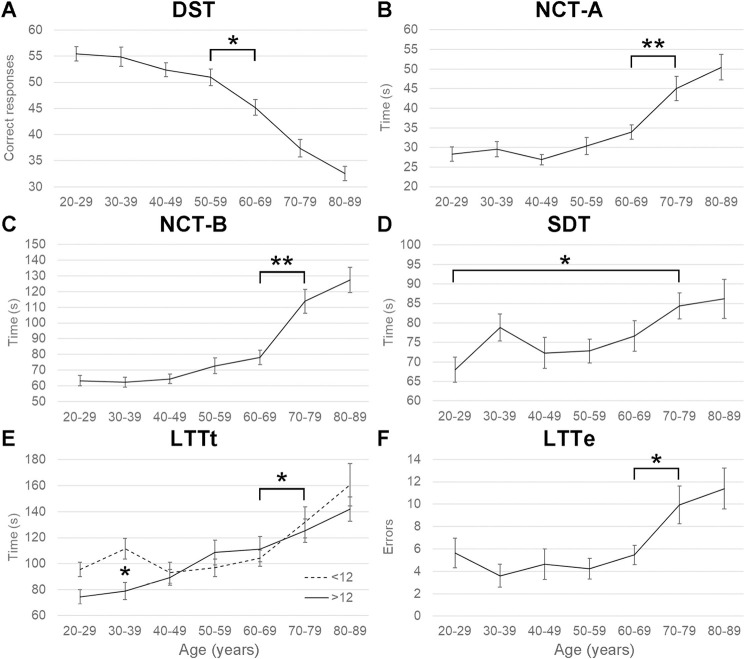
Effect of age on PHES. Plots of (A) correct responses in Digit Symbol Test (DST), time in seconds in (B) Number Connection Test part A (NCT-A), (C) NCT-B, (D) Serial Dotting Test (SDT), (E) Line Tracing Test (LTTt), and (F) errors in LLT (LTTe) on the *y* axis as a function of age levels on the *x* axis in years. Education is also illustrated in (E). Asterisks indicate significant statistical differences in Newman-Keuls post-hoc test: *, P < .05; **, P < .01.

In contrast, we evidenced a main effect of age on all measures: DST^TR^ (F(6,168) = 38.7, P < .01), NCT-A^TR^ (F(6,168) = 14.7, P < .01), NCT-B^TR^ (F(6,168) = 28.0, P < .01), SDT^TR^ (F(6,168) = 3.0, P < .01), LLTt^TR^ (F(6,168) = 14.8, P < .01), and LLTe^TR^ (F(6,168) = 6.1, P < .01). Specifically, performance declined after 60 years in DST (50 vs. 45 responses in 50–59 vs. 60–69 years, respectively; Newman-Keuls (NK) post-hoc test, P < .05; Fig 1A), whereas it declined after 70 years in NCT-A (33 vs. 45 s in 60–69 vs.70-79 years; NK, P < .01; Fig 1B), in NCT-B (78 vs. 114 s; NK, P < .01; Fig 1C), in LLTt (108 vs. 129 s; NK, P < .01; Fig 1E), and in LLTe (5.5 vs. 9.9 errors; NK, P < .05; Fig 1F). In SDT, the difference was only significant between the extreme ages (68 vs. 84/86 s in 20–29 and 70-79/80-89 years; NK, P<0.5; Fig 1D).

A main effect of education was found on DST^TR^ (F(1,168) = 11.0, P < .01), NCT-B^TR^ (F(1,168) = 18.8, P < .01), SDT^TR^ (F(1,168) = 6.6, P < .05), and LLTt^TR^ (F(1,168) = 4.8, P < .05), in which high-educated participants always performed better than low-educated ones. Education main effect was only marginal on NCT-A^TR^ (F(1,168) = 3.6, P = .06), and absent on LLTe^TR^ (F<1). Finally, only one interaction Age*Education was observed on LLTt^TR^ (F(6,168) = 2.6, P < .05), where low-educated participants aged 30–39 years performed slower than high-educated ones (Fig 1E).

### Regression analyses

**Fig 2:**
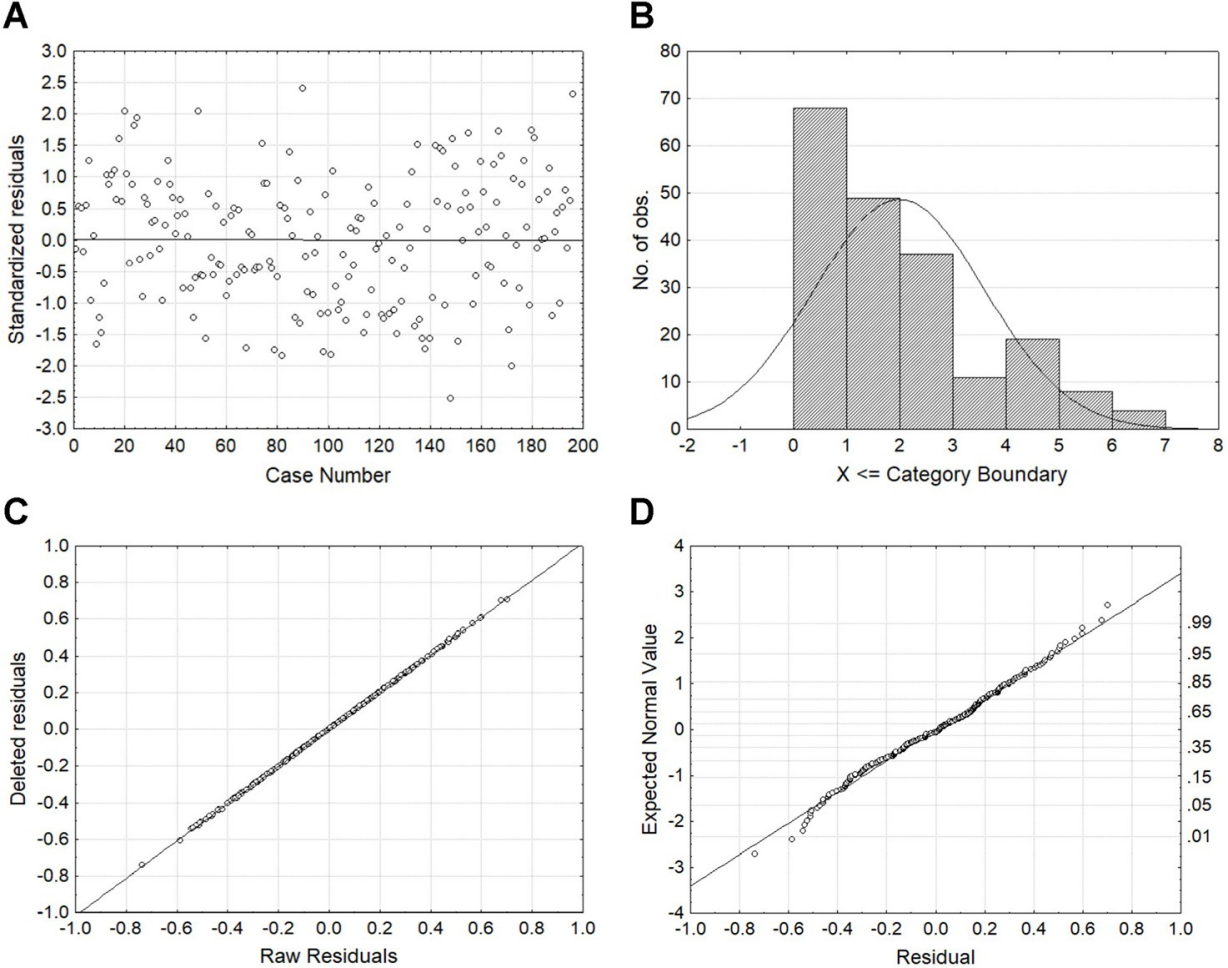
Analysis of regression outliers. Regression plots of (A) standardized residuals as a function of case numbers, (B) Mahalanobis distances, (C) deleted residuals as a function of raw residuals, and (D) expected normal values as a function of residuals, illustrated here in the Number Connection Test part B (NCT-B).

Consistent with ANOVAs, regression analyses confirmed the significant contribution of age in all measures and of education in DST^TR^, NCT-B^TR^, SDT^TR^, and LLTt^TR^. For NCT-A^TR^, education was significant (F(1,193) = 11.4, P < .01; remember it was only marginal in ANOVA) so that the factor was kept in its regression analysis. Thus, multiple regression analyses were performed for DST^TR^, NCT-A^TR^, NCT-B^TR^, SDT^TR^, and LLTt^TR^, while simple regression analysis was completed for LLTe^TR^. In each regression, we calculated the intercept, the regression slope of age, the regression slope of education (except for LLTe^TR^), and the standard deviation of residuals. As outliers may bias results, particularly in tests like SDT and LTT (see below), we completed residual analysis for each measure as illustrated in Fig 2. Case numbers vs. standardized residuals were first plot to search for cases outside plus or minus three times sigma limits (Fig 2A). Mahalanobis distances were then observed to look at those distances to identify extreme cases (Fig 2B). Raw vs. deleted residuals were also plotted so that putative outliers might pop out (Fig 2C). Finally, residuals vs. their expected normal value were also examined to check that the normality assumption was not violated (Fig 2D). Using this four-step methodology, no outlier was found for any measure.

### Normogram construction

The six transformed measures DST^TR^, NCT-A^TR^, NCT-B^TR^, SDT^TR^, LLTt^TR^, and LLTe^TR^ were retransformed into the original scale using the formula exp(*i*+(a*age)+(e*education)+(±z*s)) for the five former, and (*i*+(a*age)+(±z*s))^2^ for the latter, where *i* is the intercept, *a* the regression slope of age, *e* the regression slope of education, and *s* the standard deviation of residuals. The variable *z* is the *z*-score for five normative limits within each test: -3, -2, -1, 0, +1 for DST in which the better the performance the higher the score; -1, 0, +1, +2, +3 for the five other measures in which the worse the performance the higher the score; where 0 is the mean and ±1, 2, 3 are the normal standard deviations. The age range, which was 20–89 years in real data, was extended to 18–95 years in the implementation as participants may be younger or older in clinical practice. As in Weissenborn et al. [23], the PHES was calculated as the sum of the six subscores, which were either -3, -2, -1, 0 or +1 for respectively *z*<-3, -3<*z*<-2, -2<*z*<-1, -1<*z*<1 or *z*>1 in DST; and reversely +1, 0, -1, -2 or -3 for respectively *z*<-1, -1<*z*<1, 1<*z*<2, 2<*z*<3 or *z*>3 in NCT-A, NCT-B, SDT, LLTt and LLTe. As a result, the PHES range was -18 to +6 and the cut-off for MHE was also set to -4.

### Online implementation

French norms were implemented in a form available online that one may bookmark on a computer, tablet or mobile through the free and safe following link https://www.tnl.cnsfed.com/resource/fphes. The form is a calculator and data are not stored. Once a PHES is completed and scored, the examiner can access the form, set the participant’s age and education level, enter the six data, and immediately get the PHES. For research purpose involving a high number of participants, one may send a reasonable request to the authors.

### Relationship between French and seminal German normograms

**Fig 3:**
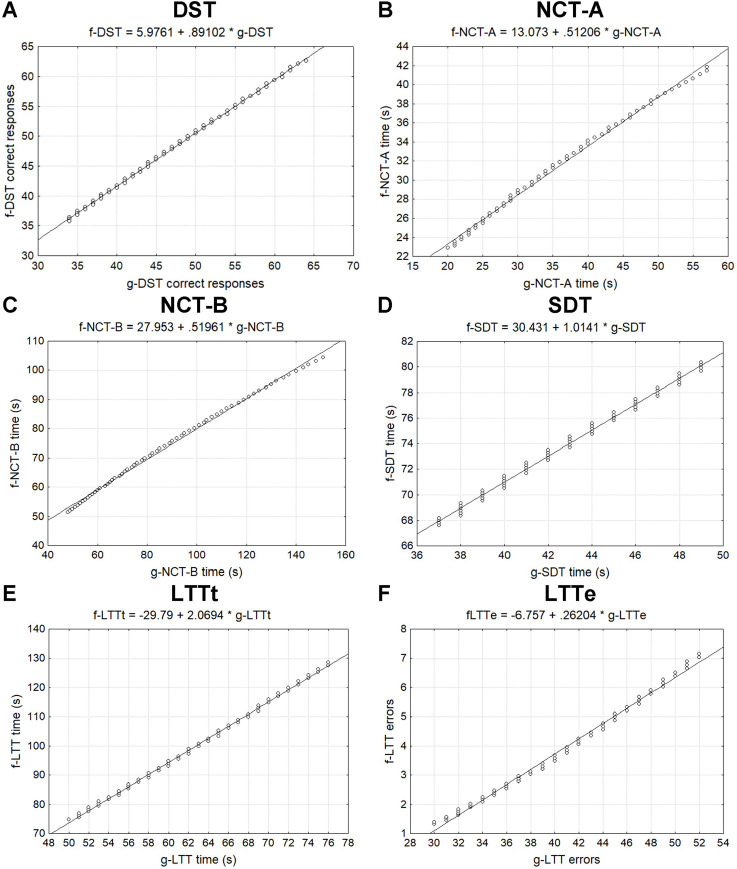
Comparison between French and seminal German norms. Correlation scatterplots of (A) correct responses in Digit Symbol Test (DST), time in seconds in (B) Number Connection Test part A (NCT-A), (C) NCT-B, (D) Serial Dotting Test (SDT), (E) Line Tracing Test (LTTt), and (F) errors in LLT (LTTe) of French (f) participants on *y* axis as a function of German (g) participants on *x* axis. Subtitles are regression equations.

To compare French and seminal German norms, we restricted the data as follows: the age range was reduced to the window of 18–80 years, the education of French data was set to the average level of 12 years, and the normative limit was set to *z* = 0. Resulting central trend and dispersion indices are shown in Table 4. French and German norms were similar for DST correct responses (Student *t* test, *t*<1), but different for other measures. Indeed, the mean was lower in French data compared to German ones for NCT-A (*t*_124_ = -2.9, P < .01) and NCT-B (*t*_124_ = -3.6, P < .01), whereas the reverse was observed for SDT (*t*_124_ = 46.7, P < .01). In LTT, French trend was slower than German one (*t*_124_ = 16.6, P < .01) but with less errors (*t*_124_ = -42.7, P < .01). Pearson analysis showed significant correlation for all measures (P < .01) as indicated by correlation and determination coefficients in Table 4 and as illustrated by scatterplots in Fig 3.

**Table 4:** Relationship between French and seminal German norms.

	DST resp.	NCT-A (s)	NCT-B (s)	SDT (s)	LTTt (s)	LTTe
French	48.0±7.9	31.5±5.6	74.8±15.6	73.8±3.8	99.4±15.9	3.9±1.7
German	47.1±8.9	35.9±10.9	90.1±30.0	42.8±3.7	62.4±7.7	40.5±6.6
Correlation *r* / *r^2^*	.999/.999	.998/.996	.998/.997	.997/.994	.999/.998	.997/.994

Mean ± standard deviation of Digit Symbol Test (DST) correct responses, Number Connection Test parts A (NCT-A) and B (NCT-B), Serial Dotting Test (SDT) times in seconds, Line Tracing Test time in seconds (LTTt) and errors (LTTe) for ages of 18–80 years, education level of 12 years (French only), and normative limit *z* = 0 (N = 63). Correlation (*r*) and determination (*r^2^*) coefficients.

## Discussion

In this study, we standardized the PHES in a French population, examined the effects of controlled factors (sex, age, and education) on PHES performance, and compared the French norms with seminal German norms [23]. The main findings were as follows. 1) Normative data of the PHES were built up in a French healthy population, in which sex, age and education variables were controlled. 2) Sex showed no effect on any measure, whereas age influenced all measures and education impacted five of six measures. 3) Optimal balance in the factor levels led to high-quality normograms where no outlier popped out. 4) French and seminal German normograms significantly fitted though some differences could be observed. 5) Online implementation allows clinicians and researchers to easily get the PHES for French patients.

The present study was the first one in the international literature to introduce a perfect ratio of all levels within each of controlled factors. Indeed, sex, age and education levels were perfectly balanced in the number of 2 times 98, 7 times 28, and 2 times 98 participants, respectively. As a result, the whole sample size was average: whilst it was higher than that of 5 previous studies [23, 45, 49, 52, 57], it was lower than that of the other 10 studies [46–48, 50, 51, 53–56, 58]. Our rationale was to collect real performance in real people, which meant to be critical in the aging population between 65 (i.e. average retirement age) and 89 years when the performance may rapidly decline, and in low-educated people in whom the result is unpredictable in psychomotor tests as soon as it is not tested. On the one hand, such strategy has the drawback of taking time, namely two years and three months, to find out the ad hoc number of healthy participants in each level of sex, age, and education. On the other, it offers real data that should be accepted as they are, rather than extrapolating putative data for conditions that have never been apprehended in real life.

Our study confirmed that sex does not influence PHES performance consistent with fourteen previous studies [23, 45–52, 54–57]. Only one study in Romania reported sex effect, which was observed in only two tests, DST and SDT [53], while Allampati et al. in USA [58] adjusted their norms to sex in addition to age and education. Our study also corroborated the systematic effect of age on all or almost all PHES measures as previously reported by all fifteen studies (see Introduction). The added-value of the present study was to refine the PHES evolution above 65 years in young old (60–69 years), middle old (70–79 years) and very old (80–89 years) healthy participants, which was not or little explored in the past [48, 54]. Our observation that education influenced most but not all PHES measures is also consistent with all previous studies, except the study in Germany which did not report this factor [23]. Taken together, age and education are crucial variables to control when interpreting the PHES outcome in patients, while the way sex may influence PHES performance in patients, particularly the oldest ones, needs to be further investigated (see Introduction).

Despite its complexity, the PHES also shows stability across the different countries where it is standardized. Our results were tightly linked to German data though some specificities exist. Two tests, DST and NCT-A, were particularly robust as our results did not or little vary from German data. In contrast, NCT-B, SDT and LTT were versatile and, as a matter of fact, the opportunity to reveal differences between the different cultures and/or cognitive styles. For example, French participants were slower in NCT-B as compared to German people. This was even more obvious in SDT and LTT in which French participants were slow to make few errors. In other words, though the instruction was the same, German participants were liberal by performing faster with errors whereas French participants adopted a conservative strategy by being slow and accurate. Those differences point out the need of building up specific normative data in each country and, eventually, in each province or state of wide countries such as China, India, Russia and USA. Within a country and across ages, some differences may also be observed beyond life cycle considerations, as was the case of our generation of low-educated participants in their thirties who adopted the slowest and most accurate strategy in LTT.

The PHES success also relies on its ability to assess all and only these cognitive functions that are impacted by MHE (see Introduction). By focusing on only one cognitive aspect, other tools may either overlook or exaggerate a given function or the significance of its success or failure. Additionally, there is a need for physicians, should they be hepatologists and neurologists, and neuropsychologists to collaborate further. For example, the EncephalApp Stroop test [34] not only focuses on attention control but is also not optimally conceived. Indeed, the test involves two inhibitory processes: the traditional inhibitory process described by Stroop [31] that prevents reading (i.e. controlled attention) to name the color (i.e. selective attention). But it also involves inhibition of return [64]: because the location of color names changes at each trial, there is a need to inhibit the previous spatial location of a given name (controlled attention) to consider its new location on every trial (selective attention). Such spatial inhibition adds complexity to the test, which may lead to failure in conditions such as fatigue [65], medication [66], normal aging [67] or any pathological prefrontal condition [68]. In other words, computerized tests such as the Inhibitory Control Test [32], the Cognitive Drug Research battery [33], the EncephalApp Stroop test [34], and the Continuous Reaction Time [35] may be more useful to monitor MHE over time or after interventions than to detect MHE.

Combining hepatology with fine neuropsychology may also contribute to further understanding MHE etiopathogenesis (see Introduction). For the neuropsychologist expert, MHE is a nonverbal syndrome which concerns three areas of cognition: first, the cognitive control of the attention and executive systems, indifferently named either the anterior or control system in attention models [69], or the central executive in working memory models [70], or the executive controller in executive function models [71]; second, motor coordination in its speed and accuracy processes [72]; and third, visual perception in its dorsal or occipital-parietal stream [73]. This syndrome is reminiscent with other neuropsychological nonverbal syndromes, in which white matter disconnection has recently been shown to play a critical role, such as developmental coordination disorder in children [74] and apraxia in adults [75]. Thus, the way MHE physiopathology ends in white matter disconnection in the human brain might be a promising future direction of research.

## Conclusions

We standardized for the first time the PHES in a French population and explored the effects of sex, age and education using perfectly balanced samples. Differences between countries emphasize the need to build up normative data in each country to get accurate PHES in patients.
